# Impact of Temperature, pH, Electrolytes, Approach Speed, and Contact Area on the Coalescence Time of Bubbles in Aqueous Solutions with Methyl Isobutyl Carbinol

**DOI:** 10.3390/polym17141974

**Published:** 2025-07-18

**Authors:** Jorge H. Saavedra, Gonzalo R. Quezada, Paola D. Bustos, Joaquim Contreras, Ignacio Salazar, Pedro G. Toledo, Leopoldo Gutiérrez

**Affiliations:** 1 Escuela de Ingeniería Civil Química, Universidad del Bío-Bío, Concepción 4081112, Chile; grquezada@ubiobio.cl (G.R.Q.); pdbustos@ubiobio.cl (P.D.B.); joaquim.contreras1601@alumnos.ubiobio.cl (J.C.); ignacio.salazar1801@alumnos.ubiobio.cl (I.S.); 2 Water Research Center for Agriculture and Mining (CRHIAM), Concepción 4030000, Chile; lgutierrezb@udec.cl; 3 Departamento de Ingeniería Química, Universidad de Concepción, Concepción 4030000, Chile; 4 Departamento de Ingeniería Metalúrgica, Universidad de Concepción, Concepción 4030000, Chile

**Keywords:** coalescence time, bubble, flotation, methyl isobutyl carbinol (MIBC), electrolyte-surfactant interactions, bubble stability, frother

## Abstract

The prevention of bubble coalescence is essential in various industrial processes, such as mineral flotation, where the stability of air–liquid interfaces significantly affects performance. The combined influence of multiple physicochemical parameters on bubble coalescence remains insufficiently understood, particularly under conditions relevant to flotation. This study explores the key factors that influence the inhibition of bubble coalescence in aqueous solutions containing methyl isobutyl carbinol (MIBC), providing a systematic comparative analysis to assess the effect of each variable on coalescence inhibition. An experimental method was employed in which two air bubbles were formed from identical capillaries and brought into contact either head-to-head or side-by-side, then held until coalescence occurred. This setup allows for reliable measurements of coalescence time with minimal variability regarding the conditions under which the bubbles interact. The study examined the effects of several factors: temperature, pH, salt concentration and type, bubble approach speed, contact area, and contact configuration. The results reveal that coalescence is delayed at lower temperatures, alkaline pH conditions, high salt concentrations, and larger interfacial contact areas between bubbles. Within the range studied, the influence of approach speed was found to be insignificant. These findings provide valuable insights into the fundamental mechanisms governing bubble coalescence and offer practical guidance for optimizing industrial processes that rely on the controlled stabilization of air–liquid interfaces. By understanding and manipulating the factors that inhibit coalescence, it is possible to design more efficient and sustainable mineral flotation systems, thereby reducing environmental impact and conserving water resources.

## 1. Introduction

The mining industry, particularly in Chile with its abundant mineral reserves, relies significantly on the flotation process for the effective extraction and recovery of valuable minerals [1,2,3]. Flotation is a complex physicochemical process that separates valuable mineral particles from gangue materials based on their differing surface properties [4,5,6,7]. While many factors influence the efficiency of flotation, the time required for bubble coalescence remains a central concern, as it directly impacts the stability of bubbles in the liquid phase and the froth layer, ultimately affecting the recovery of valuable minerals [8,9,10,11].

Bubble coalescence is a key phenomenon in the flotation process, which involves the merging of two adjacent air bubbles to form a single larger bubble [12]. The time required for this coalescence, referred to as bubble coalescence time or contact time until coalescence, can significantly affect mineral recovery efficiency and overall flotation performance [13,14]. If the coalescence time is too short, the rapid formation of larger bubbles can destabilize the froth layer, reducing the surface area available for interaction with valuable mineral particles. This can hinder their attachment and subsequent recovery. Conversely, if the coalescence time is excessively long, it can result in a dense, slow-moving froth, which impairs operational efficiency [15,16,17].

The time it takes for bubbles to coalesce is a complex phenomenon influenced by multiple factors [17,18]. Key factors include the type and concentration of flotation reagents [19,20], as well as pulp properties such as pH, temperature, and solids concentration [21]. Additionally, process water quality can significantly affect coalescence time [17,22,23]. Operational parameters, including the air injection rate, bubble distribution system, and the design and operation of flotation cells, are also crucial factors [24].

The existing literature on bubble coalescence time reveals various interconnected findings, highlighting the inherent complexity of the phenomenon. A review of several studies identifies different influencing factors, including the chemical composition of solutions and the speed at which bubbles approach each other. In a survey by Bournival et al. [25], it is suggested that a significantly higher concentration of salt (NaCl) than the frother (MIBC) is necessary to extend bubble coalescence time. This finding is further elaborated by Castro et al. [26], who indicate that a mixture of seawater and frothers can effectively stabilize bubbles and prevent coalescence, showing a more pronounced effect than when only electrolytes like NaCl are used. Additionally, Guo et al. [11] contribute to these findings by demonstrating that the type of salt used can significantly impact bubble coalescence. They showed that sodium halides can inhibit coalescence, with the effectiveness ranking as follows: NaCl > NaBr > NaI.

According to Orvalho et al. [27,28], the approach speed of bubbles significantly influences the time it takes for them to coalesce. Specifically, higher approach speeds result in quicker coalescence. Furthermore, the same authors found that bubbles formed at a mobile interface coalesce more effectively than those generated at a static interface. Gungoren et al. [29] demonstrated that the coalescence time of bubbles can be inhibited by the application of dodecylamine hydrochloride (DAH), both in the presence and absence of potassium chloride (KCl) and sodium chloride (NaCl).

The studies conducted by Wang et al. [19] and Bournival et al. [20] enhance our understanding of how surface tension influences bubble coalescence time. They suggest that variations in surface tension can affect bubble stability and, consequently, their coalescence behavior. Additionally, research by Pan et al. [17] and Yang et al. [30] provides further insight into the role of bubble size in coalescence time. Pan et al. [17] found that the addition of PAX (Potassium Amyl Xanthate) and NaHS (Sodium Hydrosulfite), even at low concentrations, significantly increases bubble lifetime while reducing bubble size. Notably, despite their effects on bubble stability and size, PAX and NaHS did not significantly alter surface tension within the concentrations studied. Yang et al. [30] examined how different types of bubble collisions can affect coalescence. While they acknowledged that temperature influences bubble coalescence, the specific extent and nature of its effect are still poorly understood and warrant further investigation. Recent studies have further advanced this understanding by investigating ion-specific effects on bubble coalescence [31], the role of interfacial mobility and pH in modulating film stability [32], and the combined influence of frothers, collectors, and electrolytes on bubble lifetime [10]. The dynamic adsorption of nonionic surfactants such as MIBC on bubble interfaces, and its impact on film drainage and coalescence time, has been analyzed both experimentally and through modeling [33]. Additionally, the influence of hydrodynamic factors, including approach velocity and confinement effects in microchannel geometries, has been quantitatively characterized [34].

The interfacial phenomena investigated in this study, including film drainage, surfactant–ion interactions, and stabilization against coalescence, are not only central to the froth phase stability in flotation systems but also relevant to a broader range of polymer-containing dispersed systems, such as foams, emulsions, and colloidal suspensions. Recent studies demonstrate that polymers can significantly alter bubble–bubble and bubble–particle interactions in flotation by modifying interfacial rheology and film drainage dynamics [35,36]. Polymers can adsorb at fluid interfaces, increasing interfacial viscoelasticity, and resisting film thinning, which contributes to the formation of more persistent and stable foams [37,38]. Molecular dynamics simulations have confirmed that polymer–surfactant–ion interactions influence drainage rates and the structuring of interfacial films under varying pH and salinity conditions [39]. Complementary experimental techniques, such as interferometry and reflectometry, have shown that polyelectrolyte/surfactant systems can form elastic interfacial layers that delay or inhibit bubble coalescence [40,41]. These findings highlight the wider applicability of the interfacial mechanisms studied here, not only to polymer-stabilized flotation systems but also to a range of soft matter applications involving interfacial stability.

This research aims to improve the understanding of bubble coalescence time and its relationship to bubble stability in aqueous systems containing methyl isobutyl carbinol (MIBC), a neutral, short-chain, nonionic alcohol used as a low-activity surfactant, commonly referred to as a frother in flotation processes. Through a systematic experimental study using a capillary-based bubble generation device, we investigated how various factors affect coalescence time, including MIBC concentration, temperature, pH, NaCl concentration, salt type, approach speed, and bubble contact area. Unlike previous studies focusing on isolated variables, this work provides a comprehensive comparative assessment of multiple physicochemical and hydrodynamic factors influencing bubble interactions under controlled experimental conditions. By clarifying the mechanisms that govern bubble stability, this study aims to enhance the understanding of the processes that control mineral flotation, ultimately leading to more efficient and sustainable recovery strategies.

## 2. Materials and Methods

### 2.1. Reagents

Deionized water was used to prepare all aqueous solutions and to clean the glassware and experimental equipment. Methyl isobutyl carbinol (MIBC, >98% purity, Merck, Germany) was used as the flotation frother and prepared at concentrations of 10, 20, 50, 100, 200, 500, and 1000 ppm. The salts used to prepare the electrolyte solutions included sodium chloride (NaCl), potassium chloride (KCl), potassium iodide (KI), magnesium chloride hexahydrate (MgCl_2_·6H_2_O), and calcium chloride dihydrate (CaCl_2_·2H_2_O). All salts were of analytical grade (Merck, Germany). Stock solutions of 1.0 M concentration were prepared for each salt. pH adjustments were carried out using hydrochloric acid (HCl, 37%) and sodium hydroxide (NaOH, pellets), both of analytical grade (Merck, Germany). All solutions were freshly prepared immediately before each experiment to minimize contamination and degradation. No additional additives, surfactants, or modifiers were included in the solutions.

### 2.2. Experimental Conditions

All measurements were performed under a set of baseline conditions, serving as the reference state for evaluating each experimental variable. Unless specified otherwise, the experiments were carried out at a temperature of 20 ± 0.5 °C, at the natural pH of the solution (~6.5), and without the addition of salts. The experimental setup employed two horizontally aligned stainless steel needles to generate air bubbles, with a bubble approach speed of 8.5 mm/s and an average contact area of about 0.46 mm^2^ between the bubbles.

The following parameters were systematically varied from the base conditions to investigate their effects on bubble coalescence time. First, the temperature was adjusted, with experiments at 15 °C and 25 °C in addition to the base temperature of 20 °C. The solution pH was modified to values of 5.0 and 9.0 using hydrochloric acid (HCl) and sodium hydroxide (NaOH), respectively. The salt concentration was altered by adding NaCl at concentrations of 0.1, 0.5, 1.0, and 2.0 M. To examine ion-specific effects, NaCl was substituted with other salts—KCl, KI, MgCl_2_·6H_2_O, and CaCl_2_·2H_2_O—maintaining a fixed concentration of 1.0 M.

**Figure 1:**
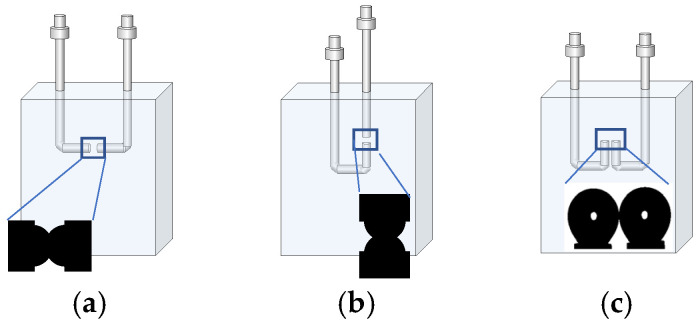
Schematic representation of contact configuration of two bubbles: (**a**) head-to-head horizontal alignment, (**b**) head-to-head vertical alignment, and (**c**) side-by-side parallel alignment.

Next, the contact area between bubbles was adjusted by changing the bubble size, reducing the contact area from approximately 0.46 mm^2^ to 0.12 mm^2^. Three bubble contact configurations were tested: head-to-head horizontal (the base case), head-to-head vertical, and side-by-side parallel (Figure 1). In horizontal and vertical head-to-head configurations, the tips of the two flat stainless-steel needles were separated by 2.0 mm, ensuring consistent bubble approach trajectories. In these head-to-head arrangements, the contact area was estimated through image analysis by measuring the diameter of the circular region formed at the interface, assuming the axial symmetry based on the captured images. This approach provided consistent and representative estimates across multiple replicates. In the parallel configuration, bubble contact was achieved by adjusting the bubble volume to promote lateral contact between them. While we aimed to achieve a qualitatively similar contact area, the geometry of the interface was less predictable due to asymmetry and shape variability. As the concentration of MIBC increased, the bubbles became more elongated, which further complicated the control of the contact geometry. For these reasons, an accurate estimate of the contact area could not be made.

Lastly, the bubble approach speed was decreased from 8.5 mm/s to 1.5 mm/s to assess the influence of hydrodynamic conditions.

### 2.3. Measurement of Bubble Coalescence Time

**Figure 2:**
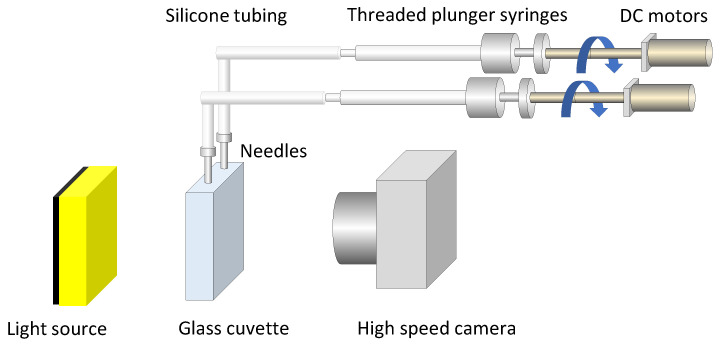
Schematic of experimental setup for generating bubbles and measuring coalescence. System consists of independently actuated syringes, motorized screw-thread plungers, stainless steel needles, and a high-speed imaging device. The arrows represent the direction of plunger motion for air injection, and the different colors are used to visually distinguish the system components.

Coalescence times were measured by recording the interaction between two air bubbles generated from two horizontally aligned stainless steel needles. These needles were submerged in a rectangular glass cuvette (20 mm × 40 mm × 80 mm) filled with the test solution. The needles, which had a flat point with an inner diameter of 1.9 mm and an outer diameter of 2.1 mm, were independently connected to 500 μL threaded-plunger glass syringes (Model 1750 LT, Hamilton Company, Reno, NV, USA) using silicone tubing with an inner diameter of 1 mm and an outer diameter of 3 mm. Air injection was controlled by motorized screw-thread plungers driven by independent reversible DC motors (Figure 2). Before each measurement, the cuvette and needles were primed with the corresponding solution, and 200 μL of air was injected through each needle. This process expelled any residual liquid, ensuring clean bubble formation. The horizontal distance between the needle tips was set to 2.0 mm.

**Figure 3:**
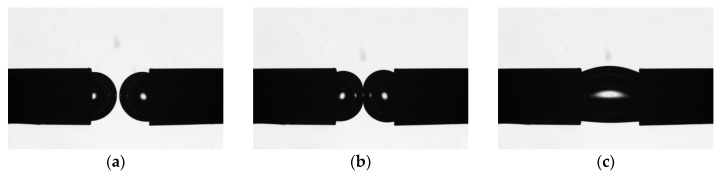
Sequence of coalescence process between two bubbles generated from horizontally aligned needles: (**a**) bubble growth and approach, (**b**) first contact between bubbles, and (**c**) complete coalescence.

The interaction between bubbles was recorded using the integrated high-speed camera of a Theta Lite TL 200 optical tensiometer (Biolin Scientific AB, Gothenburg, Sweden) operating at 66 frames per second. Coalescence time was defined as the elapsed time between the first visible physical contact of the two bubbles and their complete fusion into a single larger bubble. Figure 3 illustrates the sequence of bubble interactions: bubbles growing and approaching (Figure 3a), making first contact (Figure 3b), and completing fusion (Figure 3c).

To minimize the influence of interfacial aging or MIBC adsorption before the bubbles contact, all experiments kept the time elapsed between bubble formation and first contact between 1 and 3 s. This control ensured that the measured coalescence times primarily reflected the effects of the controlled variables rather than uncontrolled surface aging phenomena. The reported coalescence times correspond to the average of three independent replicates, each based on 20 recorded coalescence events. The error bars shown in the results figures represent the standard deviation calculated from the means of the three replicates.

## 3. Results and Discussion

The following sections present the results for each variable studied to understand their influence on bubble coalescence time. Unless otherwise indicated, all experiments were conducted under base conditions: a natural solution pH of approximately 6.5, a temperature of 20 ± 0.5 °C, horizontal head-to-head contact, a bubble approach speed of 8.5 mm/s, and an initial contact area of roughly 0.46 mm^2^. Variations from these base conditions are indicated in the corresponding subsections.

### 3.1. Temperature

**Figure 4:**
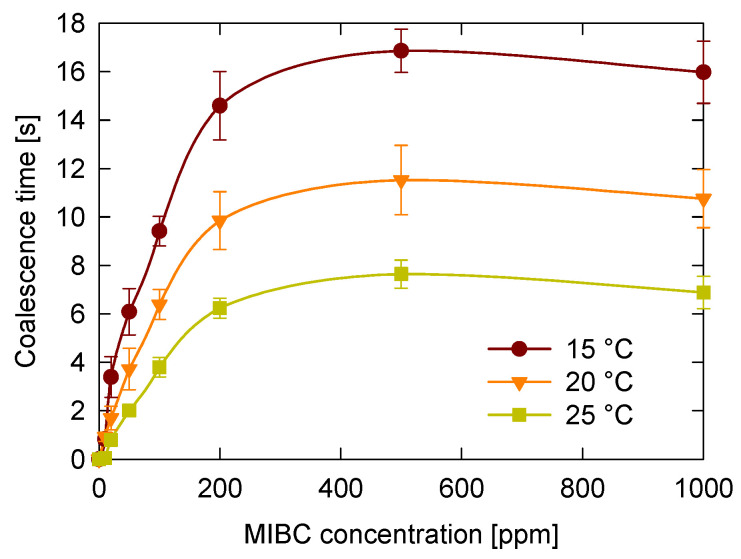
Temperature effect on coalescence time of bubbles at different MIBC concentration.

The first variable studied was temperature, which can significantly vary in industrial flotation processes. To evaluate its effect, coalescence time was measured for MIBC concentrations ranging from 0 to 1000 ppm and temperatures from 15 °C to 25 °C. The results presented in Figure 4 show that coalescence time exhibits a consistent pattern relative to MIBC concentration, regardless of temperature. It starts at very low levels, nearly zero, and sharply increases up to approximately 200 ppm. For example, at 20 °C, coalescence time rises from close to zero to about ten seconds. Between 200 and 500 ppm, the increase continues but becomes more gradual. Beyond 500 ppm, coalescence time levels off to a plateau, and at the highest concentration tested, 1000 ppm, there is a slight decrease in coalescence time.

It was found that, regardless of the MIBC concentration, higher temperatures lead to shorter coalescence times. Although temperature and frother concentration are known to reduce surface tension [26,42], this reduction does not correlate with increased bubble stability and longer coalescence times. These findings indicate that surface tension, while important for interfacial phenomena, is not sufficient to predict coalescence behavior.

These results can be explained by considering how temperature influences molecular motion and interfacial stability. At higher temperatures, molecular motion increases in both the liquid and gaseous phases, enhancing interfacial activity and resulting in more frequent molecular rearrangements. This elevated kinetic energy accelerates the transport of air, water, and MIBC molecules to and from the interface, promoting faster thinning and eventual rupture of the liquid film between bubbles [42,43]. Therefore, shorter coalescence times are observed at elevated temperatures. In contrast, at lower temperatures, molecular motion is reduced, and the viscosity of the medium is higher, which slows down the transport of frother molecules to and from the interface. This situation favors the persistence of MIBC at the interface, enhancing its stabilizing effect and leading to longer coalescence times.

### 3.2. pH

**Figure 5:**
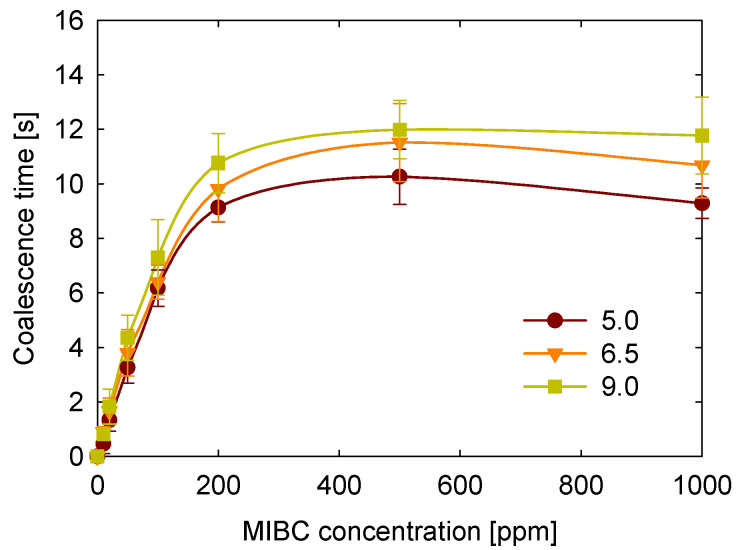
Effect of pH on coalescence time as a function of MIBC concentration.

Given the current challenges in flotation, particularly when using saline water to create a buffer effect [44], it is important to examine the impact of pH on coalescence time. Figure 5 illustrates the study of coalescence time at three different pH values in salt-free MIBC solutions to isolate the effect of pH. The results indicate a consistent trend across different pH levels: a rapid increase in coalescence times within the 0 to 200 ppm MIBC concentration range, followed by a slight decrease at higher concentrations.

Regarding the pH effect, coalescence times increase as pH increases. Since MIBC is a neutral alcohol with a pKa well above 17, it does not undergo protonation or deprotonation within the pH range of 5–9. Thus, the observed pH effect must be attributed to the ionic species used to adjust pH: HCl and NaOH.

A computational study by Mucha et al. [45] demonstrated that H_3_O^+^ and Cl^−^ ions exhibit interfacial adsorption in HCl solutions. In contrast, Na^+^ and OH^−^ remain predominantly in the bulk phase in NaOH solutions. Thus, under acidic conditions, H_3_O^+^ and Cl^−^ can occupy interfacial sites and compete with MIBC for adsorption, reducing the stabilizing effect of MIBC, and thus decreasing coalescence time. In contrast, under alkaline conditions, OH^−^ and Na^+^ interfacial exclusion allows MIBC to populate the interface more effectively, enhancing bubble stability. This behavior highlights the importance of ion-specific interfacial adsorption in influencing bubble stability, even when the MIBC itself is chemically neutral.

### 3.3. NaCl Concentration

**Figure 6:**
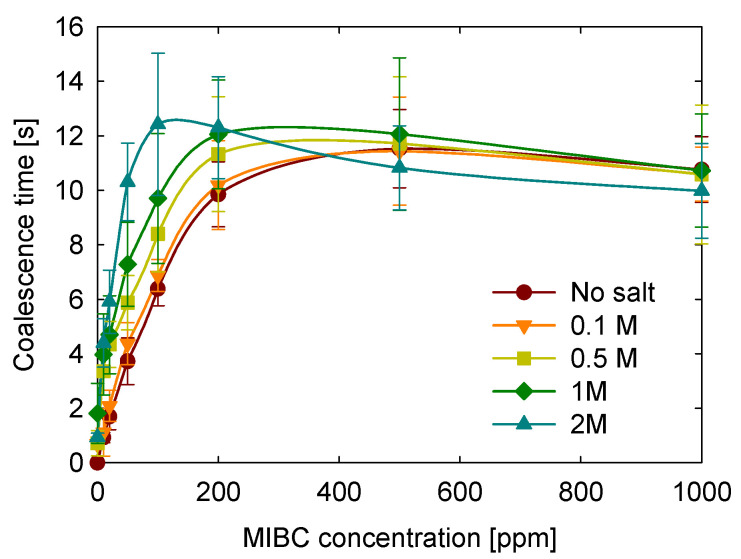
Effect of NaCl concentration on coalescence time as a function of MIBC concentration.

The effect of NaCl concentration on bubble coalescence was studied over a range from 0 to 2 M, representing conditions from freshwater to brine. As shown in Figure 6, the coalescence time increases with salt concentration from 0 to 1 M across a wide range of MIBC concentrations. For MIBC concentrations between 0 and 100 ppm, all salt levels produce an increase in coalescence time. This trend agrees with previous studies [22,46] and is often attributed to the accumulation of anions (particularly Cl^−^) at the negatively charged bubble interface, which enhances electrostatic repulsion and delays film rupture by forming a diffuse electrical double layer.

In addition to these electrostatic effects, high salt concentrations may facilitate the transfer of MIBC molecules from the bulk solution to the interface. This increased tendency of MIBC to adsorb at the interface, even at low concentrations, could lead to greater surface coverage and contribute to bubble stabilization. Although the surface may not yet be saturated, the increased presence of MIBC at the interface likely works in synergy with ionic effects to prolong coalescence time. Moreover, salt-induced restructuring of the interfacial water layer, which is known to increase its rigidity, may further reduce drainage and coalescence between bubbles.

For intermediate MIBC concentrations (200–500 ppm), the influence of salt becomes less pronounced. Notably, the curve for 2 M NaCl is lower than for all other conditions, including the no-salt case, suggesting a reversal in the stabilizing effect. This behavior indicates a more complex interfacial scenario where MIBC and ions coexist and compete for adsorption at the interface. As shown in previous studies [47], high salt concentrations can drive MIBC molecules toward the interface by altering bulk solubility and interfacial organization. However, in these crowded conditions, partial desorption of ions may occur, weakening the electrostatic stabilizing effect and ultimately reducing coalescence times.

At MIBC concentrations above 500 ppm, all curves converge regardless of salt concentration, indicating that the interface is saturated. This behavior is consistent with previous studies that report interfacial saturation at similar concentrations. Our results further indicate that coalescence times remain unchanged despite increasing MIBC concentration, suggesting that the interface may be saturated with MIBC.

### 3.4. Salt Type

**Figure 7:**
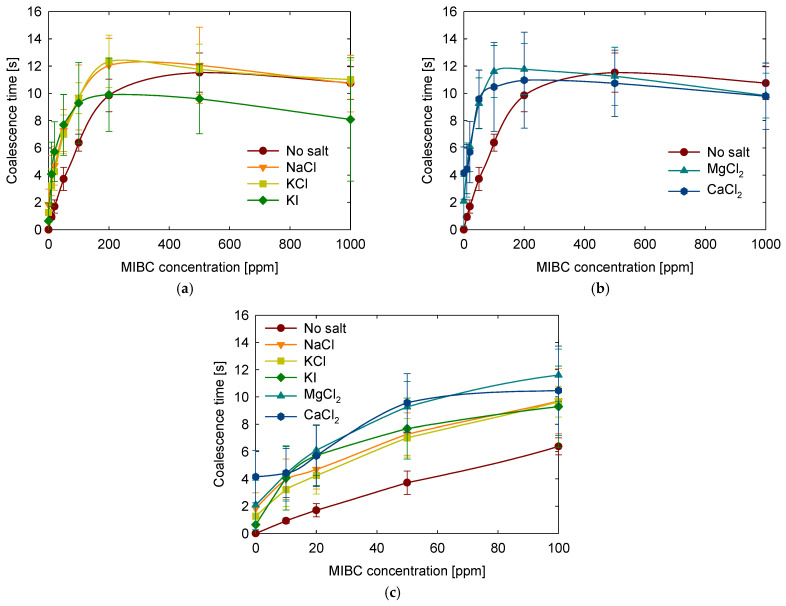
Effect of salt type on coalescence time as a function of MIBC concentration. (**a**) Monovalent salts, (**b**) divalent salts, and (**c**) a close-up view in range of 0 to 100 ppm for all salts.

Following the evaluation of how salinity influences bubble coalescence, the focus shifted to understanding how the specific identity of dissolved ions affects this behavior. In particular, the roles of cation and anion types, as well as ionic valence, were examined by comparing five salts at a fixed concentration of 1 M: NaCl, KCl, and KI (monovalent salts), and MgCl_2_ and CaCl_2_ (divalent salts). The results are shown in Figure 7.

All tested salts increased coalescence time at low MIBC concentrations and slightly decreased coalescence time at high MIBC concentrations. This general trend aligns with previous findings that attribute this behavior to the influence of electrolytes on the interactions between bubbles. It has been suggested that salts reduce gas solubility and weaken bubble–bubble attraction through a mechanism known as the salinization effect [48]. Other interpretations draw from classical DLVO theory, where bubbles are treated as interacting charged entities, and ions adsorbed at the surface contribute to film stabilization [49].

Examining the monovalent salts in Figure 7a shows that NaCl, KCl, and KI produce similar effects up to around 200 ppm of MIBC. In this concentration range, coalescence time increases in the presence of any of the three salts. However, above 200 ppm, the trends begin to diverge. While NaCl and KCl maintain a modest stabilizing effect, adding KI significantly reduces coalescence time. This suggests that the nature of the anion plays a critical role in interfacial behavior. According to molecular dynamics simulations [47], cations have limited direct influence, and interactions with the anions and the hydroxyl group of MIBC largely governs their behavior at the interface. The contrasting behavior of KI, particularly at higher MIBC concentrations, highlights the stronger destabilizing effect of the iodide ion, which may interfere with interfacial organization and MIBC adsorption, possible due to the larger size and higher polarizability of the iodide ion. This is supported by previous experimental observations indicating that KI disrupts bubble shape and promotes coalescence [50].

Figure 7b shows the results for the divalent salts MgCl_2_ and CaCl_2_. At low to moderate concentrations of MIBC (up to approximately 200 ppm), divalent salts generally offer better stabilization than monovalent salts. However, at higher MIBC concentrations (ranging from 500 to 1000 ppm), the differences between the types of salts become less pronounced. This behavior can be attributed to the higher ionic strength and stronger structuring effects of divalent cations, which enhance electrostatic screening and modify interfacial water properties [18]. These findings are consistent with previous studies showing that multivalent salts are more effective in delaying coalescence at low surfactant concentrations [51].

At low MIBC concentrations, particularly in the range of 0 to 100 ppm presented in Figure 7c, a clear trend is observed in which divalent salts (CaCl_2_, MgCl_2_) provide longer coalescence times than monovalent salts (NaCl, KCl, KI), and all salts exhibit a stabilizing effect compared to pure water. This general behavior reflects the influence of ion-specific effects, likely related to ion-induced structuring of interfacial water layers that enhance film stability by increasing interfacial rigidity and slowing drainage.

In the 500 to 1000 ppm MIBC range, a general decline in coalescence time is observed for all salt types. In this high-concentration regime, the influence of the ions becomes less significant due to the dominant effect of MIBC at the interface. The MIBC saturates the available interfacial area, diminishing the differences between the salts. Interestingly, NaCl and KCl show greater resistance to this drop in stability, while the presence of KI, MgCl_2_, and CaCl_2_ is associated with a more pronounced reduction in coalescence time. This suggests that larger or more polarizable anions, such as iodide, may hinder MIBC adsorption or be more readily desorbed from the interface when surface coverage becomes high. As a result, interfacial packing may be less efficient, leading to reduced film stability and earlier rupture, as previously described for iodide-containing systems [50].

### 3.5. Contact Area Between Bubbles

**Figure 8:**
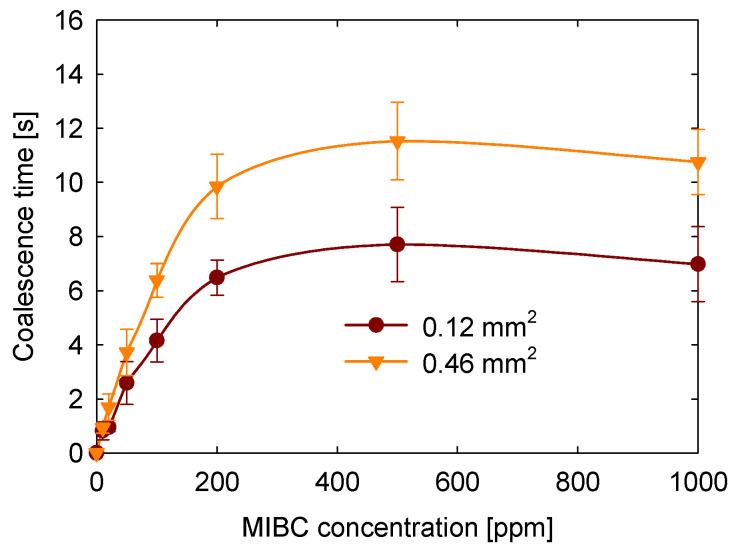
Effect of contact area on coalescence time as a function of MIBC concentration.

The effect of the contact area between bubbles on coalescence time was evaluated, as shown in Figure 8. These experiments were conducted at 20 °C, without salt addition, at a bubble approach speed of 8.5 mm/s, and at the natural pH of the solution (approximately 6.5). The results show that coalescence time increases as the contact area between the bubbles becomes larger. For example, at 100 ppm MIBC, the coalescence time for the largest contact area tested (0.46 mm^2^) was approximately 3 s longer than for the smallest contact area (0.12 mm^2^). This trend was consistently observed across all MIBC concentrations studied.

This behavior can be explained by the fact that a larger contact area results in a more extended liquid film between the bubbles, which takes longer to drain and is more resistant to rupture. Additionally, the increased surface area at the interface may enhance the adsorption of MIBC molecules, allowing for the development of interfacial concentration gradients. These gradients can induce Marangoni stresses that counteract film thinning, thus further stabilizing the interface. Although these dynamics were not directly measured in this study, it is likely that the film drainage dynamics and interfacial elasticity contribute to the observed increase in coalescence time with larger contact areas.

### 3.6. Bubble Contact Configuration

**Figure 9:**
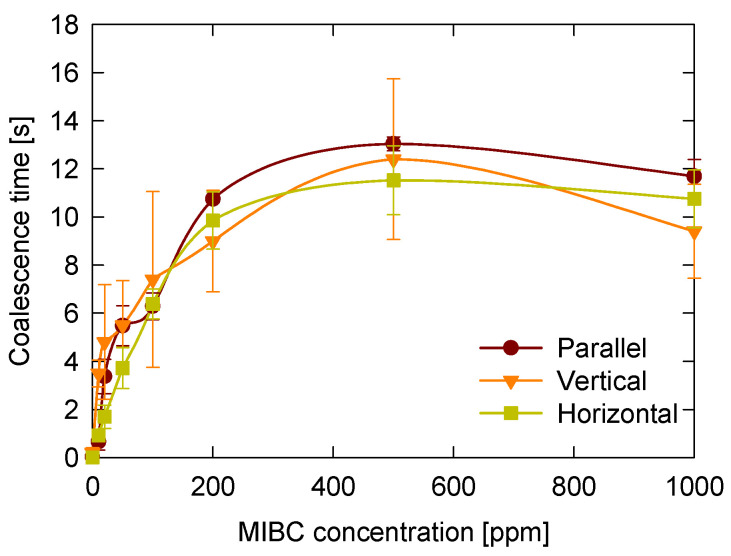
Effect of bubble contact configuration on bubble coalescence time as a function of MIBC concentration. Three configurations were evaluated: head-to-head horizontal and vertical alignment, and side-by-side parallel alignment.

The configuration of bubble contacts influences the magnitude of the coalescence time but does not significantly alter the overall trend observed as MIBC concentration increases. Figure 9 shows how coalescence time varies with MIBC concentration for three different contact configurations: head-to-head horizontal, head-to-head vertical, and side-by-side parallel.

In all configurations, coalescence time increases with MIBC concentration, reaching a plateau between approximately 200 and 400 ppm, followed by a slight decrease at higher concentrations (1000 ppm). The general shape of the curves is similar across all three configurations, indicating that the effect of MIBC adsorption dominates the coalescence behavior.

However, the side-by-side parallel configuration consistently exhibits higher coalescence times, particularly within the plateau region. This behavior may be attributed to a larger and less controlled contact area, as well as to more pronounced bubble deformation during growth and approach in this configuration, resulting in increased sensitivity to interfacial properties. As previously discussed, the contact area in head-to-head configurations was consistently estimated based on axial symmetry. In contrast, the lateral nature and geometric variability of the side-by-side configuration hindered reliable quantification.

The differences between the head-to-head horizontal and vertical configurations were relatively small regarding average coalescence time; however, the vertical arrangement showed greater variability, as indicated by larger error bars. This increased dispersion could reflect a higher sensitivity to minor asymmetries in bubble growth or alignment under vertical conditions, which could lead to fluctuations in film drainage and rupture dynamics.

In summary, while the contact configuration has a measurable effect on coalescence time, its influence is secondary to the predominant role of interfacial stabilization provided by the MIBC.

### 3.7. Approach Speed Between Bubbles

**Figure 10:**
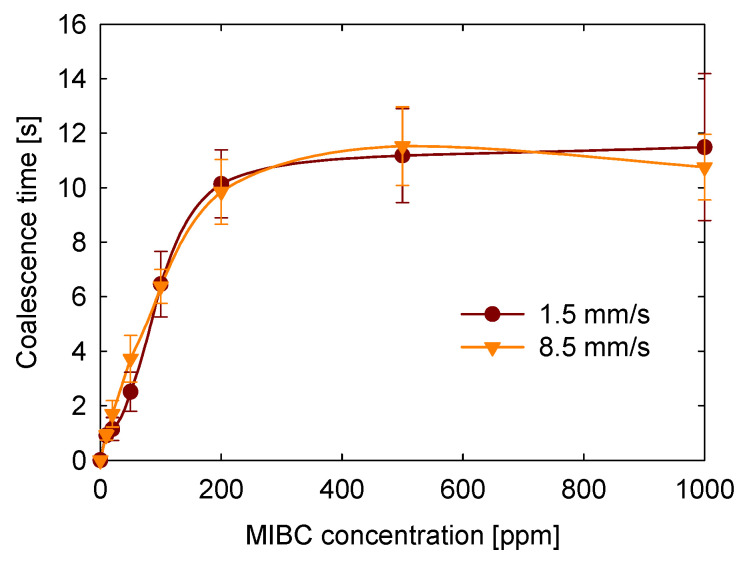
Effect of approach velocity on coalescence time as a function of the MIBC concentration.

Another objective of this study was to evaluate how the approach velocity of bubbles affects coalescence time. Figure 10 presents the results for two tested velocities: 1.5 and 8.5 mm/s. Within this range, no statistically significant differences in coalescence time were observed. This result is expected because, at low speeds, film drainage between bubbles should be governed predominantly by capillary and surface forces, rather than by inertial or hydrodynamic effects associated with the approach velocity. These findings are consistent with those reported by Del Castillo et al. [22], who also found no appreciable variation in coalescence times for approach velocities between 1 and 10 mm/s. Therefore, our results confirm that, within this low-velocity regime, coalescence dynamics are dominated by interfacial properties rather than hydrodynamic factors.

## 4. Conclusions

This study systematically evaluated the effects of temperature, pH, salt concentration and type, bubble approach speed, contact area, and contact configuration on the coalescence time of bubbles in aqueous MIBC solutions. The results demonstrated that higher temperatures reduce coalescence time due to enhanced molecular mobility and interface renewal, which accelerate film drainage and rupture. In contrast, a more alkaline pH extended coalescence times, likely by reducing interfacial ionic competition, thereby favoring the adsorption of MIBC molecules. Electrolytes also had a significant impact on bubble stability. At low MIBC concentrations, an increase in NaCl extended coalescence time; however, at higher concentrations, this effect diminished due to interfacial saturation. The type and valence of the ions influenced stability as well, with divalent salts producing stronger effects than monovalent ones, and both types being more effective than salt-free solutions. These ion-specific contributions were most relevant under low MIBC concentration, where interfacial composition is more susceptible to competitive adsorption.

The geometry of bubbles significantly affected the dynamics of coalescence. Larger contact areas resulted in longer coalescence times, likely due to slower film drainage and greater interfacial deformation. In contrast, bubble approach velocity had negligible effect within the tested range. These results highlight the need for precise control of geometric parameters to isolate and interpret interfacial phenomena.

This study advances the understanding of how physicochemical and geometric factors govern bubble stability, providing insights for optimizing flotation systems in saline or recycled water. Future research should explore the combined effects of frothers, ions, and suspended solids under agitated conditions to replicate industrial environments better. The interfacial mechanisms described are also relevant to other dispersed systems, including foams, emulsions, and polymer-based dispersions.

## Data Availability

The original contributions presented in the study are included in the article, further inquiries can be directed to the corresponding authors.

## Abbreviations

The following abbreviations are used in this manuscript:

MIBC	Methyl isobutyl carbinol
